# Valproic Acid Causes Extensive Cell Detachment and Death in Differentiated Sh-SY5Y Cell Cultures: An In Vitro Observation

**DOI:** 10.7759/cureus.88284

**Published:** 2025-07-19

**Authors:** Ioannis Dardalas, Roza Lagoudaki, Efstratios K Kosmidis, Vasilios Kimiskidis, Theodoros Moysiadis, Dimitrios Kouvelas, Chryssa Pourzitaki

**Affiliations:** 1 Laboratory of Clinical Pharmacology, Medical School, Aristotle University of Thessaloniki, Thessaloniki, GRC; 2 Laboratory of Physiology, Medical School, Aristotle University of Thessaloniki, Thessaloniki, GRC; 3 Laboratory of Clinical Neurophysiology, Medical School, Aristotle University of Thessaloniki, Thessaloniki, GRC; 4 Department of Computer Science, School of Sciences and Engineering, University of Nicosia, Nicosia, CYP

**Keywords:** in vitro, neurotoxicity, sh-sy5y, valproate, valproic acid

## Abstract

Introduction

Valproic acid (VA) has been a widely used drug in the management of epilepsy, migraine headaches, and bipolar disorder for many decades. Recently, the drug has been tested in various types of malignancy, showing promising results. Despite its well-established effectiveness, there are certain safety concerns that should be taken into consideration. VA has been classified as teratogenic and should be avoided during pregnancy.

Methods

This study was designed in order to present the effects of valproate on differentiated SH-SY5Y cell cultures and their concentration dependency. SH-SY5Y cells were differentiated into a mature neuron-like phenotype and then treated with VA at either 1 mM or 10 mM. Morphological criteria were compared between treated and untreated cells, serving as a control for identification.

Results

It was observed that VA consistently induces extensive detachment of the adherent cells and cell death. At a concentration of 1 mM, a 44,0% reduction of adherent cells was observed, while at a concentration of 10 mM, the reduction was 95.9%.

Conclusion

VA causes extensive cell detachment and death in differentiated SH-SY5Y cell cultures. There is a paucity of studies on these effects, which underlines the importance of further and more extensive research on this matter.

## Introduction

Valproic acid (VA), a valuable and widely used drug, was first synthesized in 1882 but was approved as an antiseizure agent decades later [1]. Subsequently, indications like migraine prophylaxis and management of bipolar disorder followed, and more recently, VA has also been tested as an anti-cancer agent [2]. Its pharmacodynamics involve various mechanisms, even though not all of them are entirely understood, contributing to its wide therapeutic effects. VA augments gamma-aminobutyric acid (GABA) levels in the brain by inhibiting GABA transaminase, decreases excitatory neurotransmission by modulating glutamate release, while also targeting voltage-gated sodium channels and T-type calcium channels [3,4].

It has been reported that VA, being a histone deacetylase inhibitor, has shown potential as adjuvant therapy in breast cancer patients. The proposed mechanisms of its anti-cancer action are its effect on cell migration, cell proliferation, and alteration of cell survival and apoptosis [5]. VA, as an epigenetic anti-cancer drug, has also exhibited efficacy against other types of cancer and solid tumors, namely, prostate cancer, pancreatic cancer, and cholangiocarcinoma, in both clinical and preclinical studies [6,7]. VA’s cytotoxicity in neuronal models has been linked to its action as a histone deacetylase (HDAC) inhibitor, a class of potent epigenetic modulators altering histone protein acetylation [5]. It has also been observed that VA can inhibit proliferation and induce immunogenicity of cultured neuroblastoma cells. Studies have shown that it can affect cell cycle processes and programmed cell death pathways. It has also been described that VA may induce apoptosis or autophagy, as observed in prior studies using undifferentiated neuroblastoma cells [5]. However, its effects on differentiated neuroblastoma cells, which may be more sensitive to these mechanisms, remain underexplored.

Despite the significant and well-established therapeutic value of VA, it is well-known that VA exerts a plethora of adverse effects. For instance, it is associated with a wide range of congenital malformations (e.g., spina bifida and anencephaly, cardiovascular defects, cleft lip and palate), as well as neurodevelopmental deficits, such as cognitive impairment and autism spectrum disorders [8]. Consequently, VA is also strongly contraindicated during pregnancy [9]. Despite studies on VA’s effects in undifferentiated SH-SY5Y cells, its impact on differentiated SH-SY5Y cells, which exhibit mature neuronal phenotypes, including the presence of neurites, remains unknown, representing a critical gap in understanding its neurotoxic potential.

SH-SY5Y is a human-derived neuroblastoma cell line often used in neurobiology and neuropharmacology. In its differentiated state, cells present a mature neuron-like phenotype distinguished by neuronal markers [10]. This study investigates the concentration-dependent effects of VA on the morphology and viability of differentiated SH-SY5Y neuroblastoma cells at bibliographically relevant concentrations.

## Materials and methods

The human neuroblastoma cell line was purchased from Sigma-Aldrich (USA). Eagle's Minimum Essential Medium (EMEM), Dulbecco Modified Eagle’s Medium (DMEM), and fetal bovine serum (FBS) were also purchased from Sigma-Aldrich (USA). Penicillin/streptomycin (Pen/Strep), N2 supplement, trypsin-EDTA (1X), and neurobasal medium were purchased from Gibco^TM^. Glutamine (100X) was purchased from Biowest (USA), and human brain-derived neurotrophic factor (BDNF) was acquired from Abbkine (Abbkine Scientific Co., Ltd., USA). VA was used as a lyophilized powder diluted in water for injection as per the manufacturer’s guidelines (HEXAQUIN P.SV.INJ.F 400 mg/vial, Demo A.B.E.E.).

 SH-SY5Y cells were incubated in EMEM under controlled optimal conditions (5% CO_2_; 37°C) on T25 cell culture flasks, containing penicillin-streptomycin (1%), glutamine (2 mM), and FBS (15%), before being transferred to 35 mm Petri dishes (35 mm x 10 mm), until the optimal cell confluency was achieved. Subsequently, we used a two-step six-day differentiation protocol based on retinoic acid (RA) (10 μM) and BDNF (50 ng/mL (v/v)) as proposed by Foster et al., offering a widely used, highly effective, and robust experimental model of differentiation in neuroscience [11]. The phase one medium consisted of DMEM containing high glucose (25 mM) without sodium peruvate, penicillin-streptomycin (1%), 4 mM L-glutamine, and 10 μΜ all-trans retinoic acid. After three days, the phase one medium was replaced by the phase two medium. The second medium of the differentiation protocol consisted of a neurobasal medium (NB) without phenol red, 100X N-2 supplement (1%), 50 ng/mL (v/v) BDNF, 1% L-glutamine, and 1% penicillin-streptomycin. Six days after the initiation of the differentiation protocol, cells were differentiated, spread evenly on the dish surface, and showed a network of branched neurites. According to the literature, while only 2% of undifferentiated cells possess neurites, nearly 99% develop them upon reaching the differentiated state, resembling the observational characteristics described during this experiment [11].

Differentiated SH-SY5Y cells were then treated with VA at the bibliographically relevant concentrations of 1 mM and 10 mM [12,13]. Cell culture medium was carefully replaced in all petri dishes. One minute after the treatment with VA at either 1 mM or 10 mM, cell cultures were observed under an optical microscope. Cell cultures where no VA was added to their medium were observed at the same time point and served as a negative control. Multiple fields of view (five fields per cell culture) were examined to ensure a representative assessment. Cell counts were quantified by manually counting adherent cells across five randomly selected fields of view per Petri dish. Cell counts were averaged to estimate adherent cell density (cells/cm²). This process was standardized across three independent experiments to ensure consistency. Results reflect a consistent pattern observed across replicates, with percentage reductions in adherent cells calculated relative to the control group. A 10x magnification lens was used alongside a 1.5x manual auxiliary magnification, resulting in a total of 15x magnification.

The methods utilized in this study, including differentiation and microscopy protocols, were designed to ensure high reproducibility based on well-established and standardized techniques. Cell cultures and the differentiation protocol for the SH-SY5Y cells are commonly used in neuroscience to generate neuron-like phenotypes and do not require specialized techniques beyond standard cell culture procedures.

## Results

Cell cultures in the absence of VA served as negative controls. VA induced extensive cell detachment and cell death at both concentrations. Morphological criteria were compared between treated and untreated cells for identification. The results reported were derived from three independent experiments observing the same outcome and are presented in Table 1.

**Table 1:** Dose-dependent effects of valproic acid (VA) on differentiated SH-SY5Y cell cultures regarding concentrations of 1 mM and 10 mM

	Adherent cell density (cells cm^2^)	% Reduction in adherent cells compared to control	Morphology	Cell debris
Control	30.955	0.0	Well-defined cells with dense neurite networks, spread evenly	No cell debris
1 mM VA	17.325	44.0	Slightly irregular or well-defined cells with a very low-density neurite network, formation of cell clumping	Small amounts of cell debris
10 mM VA	1.274	95.9	Shrunken, apoptotic cells, absence of neurites	Cell debris

**Figure 1:**
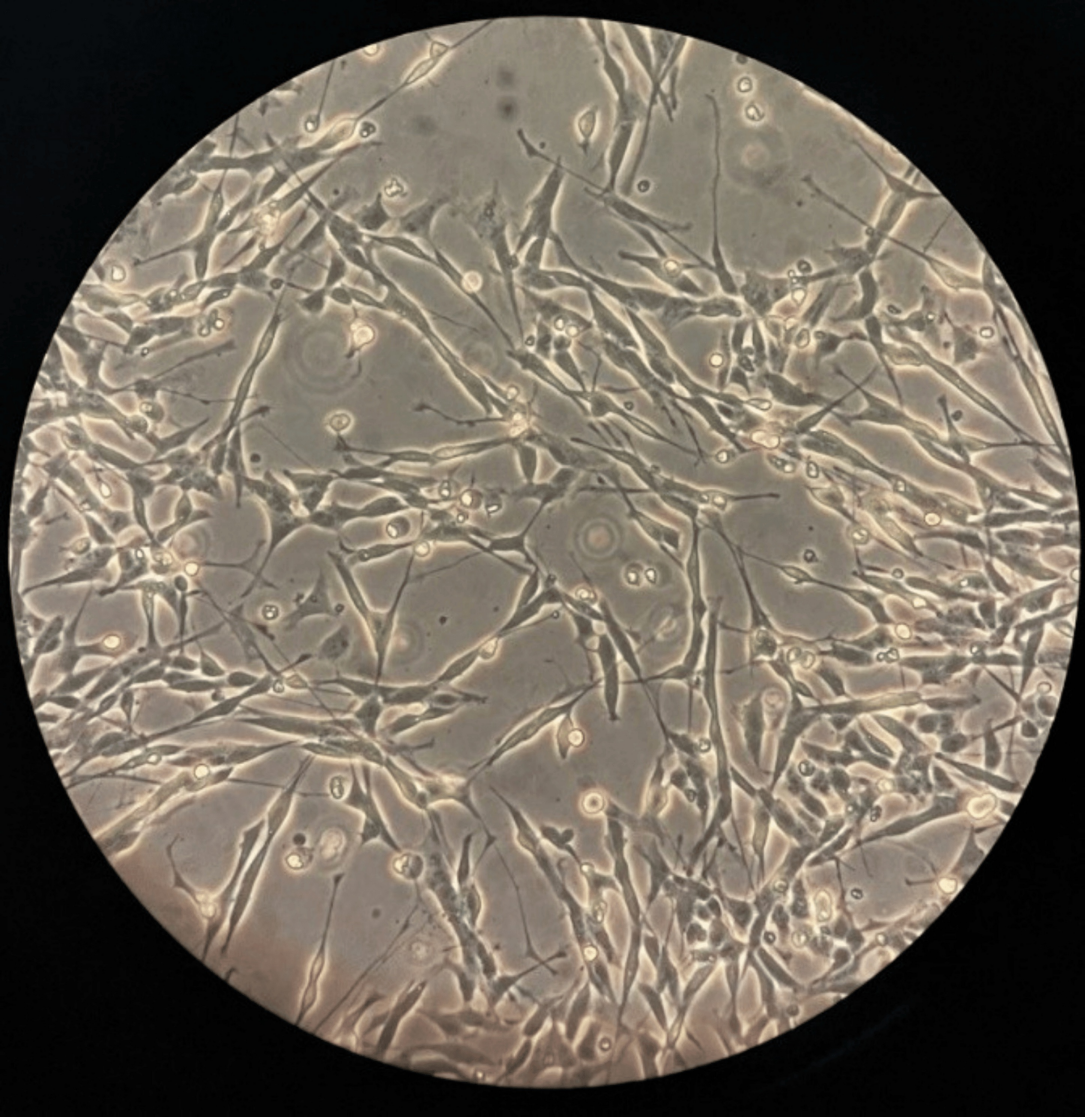
Differentiated SH-SY5Y cell culture at 15x magnification showing evenly spread cells with a dense network of elongated and branched neurites

**Figure 2:**
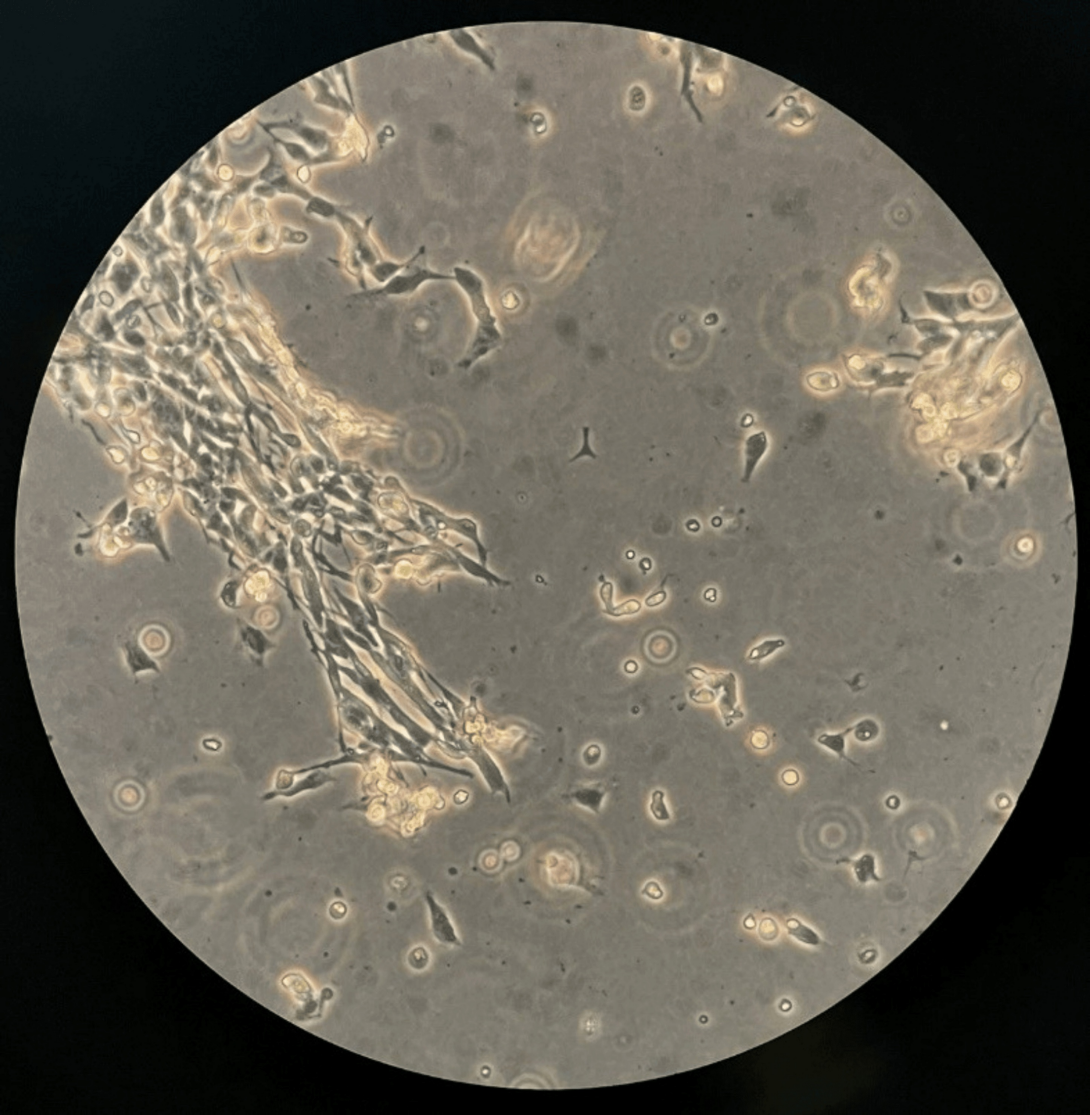
Differentiated SH-SY5Y cell culture after the addition of 1 mM VA at 15x magnification showing small amount of cell debris, reduced neurite density and formation of cell clumping

At the designated time point of one minute, the control (Figure 1) showed consistent confluency with zero change (30.955 cells/cm^2^), exhibiting a neuronal phenotype characterized by evenly spread cells with a dense network of elongated and branched neurites extending from the cell bodies, all of which appeared to be adherent to the Petri dish surface. Moreover, no cell debris was observed. At a concentration of 1 mM (Figure 2), the priorly adherent cells were suspended as a result of the addition of VA, while a proportion of cells were not observed as alive, leading to a 44.0% reduction of adherent cells (17.325 cells/cm^2^), suggesting partial cell stress or death. Small amounts of cell debris were observed, branched neurites were severely reduced, and previously evenly spread cells started forming clumps.

**Figure 3:**
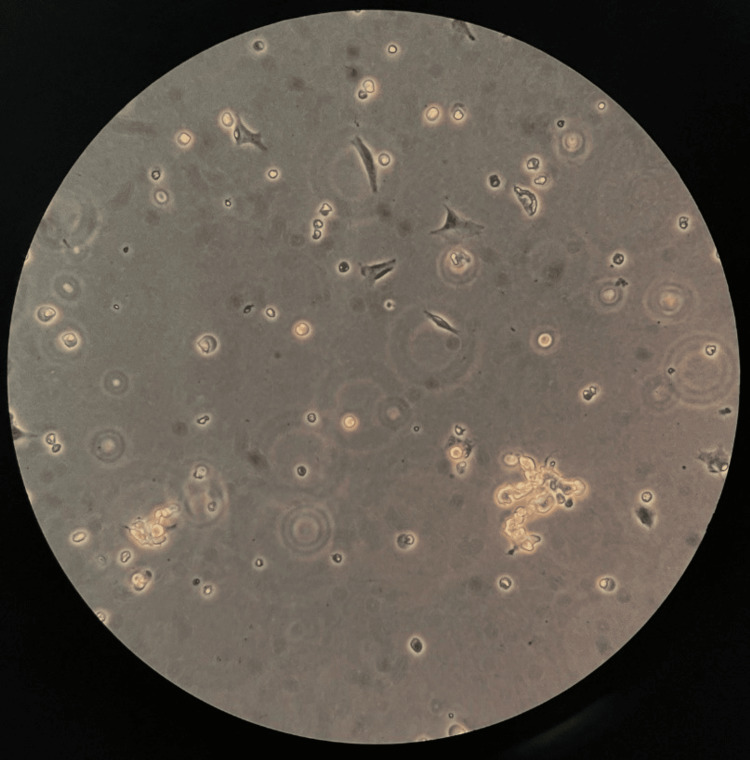
Differentiated SH-SY5Y cell culture after the addition of 10 mM VA at 15x magnification showing cell debris, complete neurite retraction, and severe cell body shrinkage, suggesting advanced apoptosis

The observations were concentration-dependent and more profound at 10 mM (Figure 3), where a 95.9% reduction of adherent cells was observed (1274 cells/cm^2^), showing severe cytotoxic effects. The majority of cells were detached, appearing as suspended clusters or fragments, leaving the Petri dish surface empty of adherent cells. The minimal remaining adherent cells exhibited complete neurite retraction, with no visible network and severe cell body shrinkage, suggesting advanced apoptosis. These observations highlight the acute and full disruption of the differentiated neuronal phenotype at this biologically relevant concentration of 10 mM VA.

## Discussion

Several studies have evaluated the effects of VA in vitro. Pacico et al. [14] used rat primary cortical cultures and found that VA dose-dependently suppressed intracellular calcium oscillations at concentrations ranging from 1 mM to 5 mM. Furthermore, a study in neuron-glia cultures showed that VA promotes neurotrophic factors release from astrocytes, thus having a neuroprotective effect on dopaminergic neurons [15].

Certain studies performed on SH-SY5Y cells have highlighted the neuroprotective and anti-apoptotic effects of VA. Pan et al. [12] reported that pretreatment with VA (5 mM) protected the SH-SY5Y cell cultures from rotenone-induced apoptosis, demonstrating a neuroprotective effect on the cells [12]. Another study on SH-SY5Y cell cultures found that VA (1-15 mM) exerts antioxidative activity on glutamate-induced excitotoxicity and increased cell viability even at the lowest assessed concentration of 1 mM [13].

 In a sharp contrast, Jang et al. [16] used VA on undifferentiated SH-SY5Y cell cultures at various concentrations (1, 5, and 10 mM) and found that VA modulates FOXO3a, a central transcription factor, and induces autophagy and mitochondrial dysfunction, while also manifesting in vitro toxicity. Thus, it emerges as a promising candidate for future studies in the management or treatment of neuroblastoma [16]. Moreover, VA appeared to inhibit the proliferation of undifferentiated neuroblastoma cells. The researchers used concentrations ranging from 0.5 mM up to 10 mM and found a time- and dose-dependent cytotoxic effect of the drug, while the concentration at which only half of the cells remained alive was established at 7.5 mM [17].

It is important to highlight that none of these studies implemented differentiated cell cultures. In our experiments, we used differentiated SH-SH5Y cell cultures, which may play a crucial role in the observed effects of the drug. There is no information in the relevant literature regarding the use of VA on differentiated SH-SH5Y cell cultures; hence, the present findings may further clarify its cellular effects. The neuron-like phenotype of differentiated SH-SY5Y cells is characterized by extensive neurite networks and expression of neuronal markers [10]. These mature neuronal cells are expected to show higher sensitivity to VA’s cytotoxic effects compared to undifferentiated cells. It has been reported that differentiated cells show higher vulnerability to oxidative stress and reduced mitochondrial membrane potential, while also different pathways are affected [11]. The rapid cell detachment and apoptosis observed in our study correlate with this hypothesis, suggesting that the differentiated cells may augment the VA’s neurotoxic effects in contrast to the neuroprotective effects reported in undifferentiated cells [12].

This study provides an observation with potential value into the effects of VA on bibliographically relevant concentrations, on differentiated neuroblastoma SH-SY5Y cell cultures, but has certain limitations. The study tested VA at 1 mM and 10 mM, which are bibliographically relevant but represent a narrow range. Additional concentration testing, especially with lower concentrations, could better elucidate the relationship between dose and response, indicating the threshold for cytotoxicity in differentiated SH-SY5Y cells. The use of a one-minute exposure time was chosen to show acute morphological changes but may not reflect chronic cytotoxicity or resemble long-term exposures, which are typical in therapeutic protocols. Furthermore, this study only observed the effects of VA on SH-SY5Y cells. While valuable for neurobiology and neurotoxicology, it may not fully represent the full diversity of neuronal anti-cancer or toxic cell responses to this drug, but including other cell lines could potentially enhance the generalizability of these observations. Moreover, while the study observed cell detachment and death, it did not investigate the underlying molecular mechanisms. Therefore, these novel findings may provide a foundation for future research to draw more comprehensive conclusions.

## Conclusions

VA is a valuable drug in the management of many diseases, including epilepsy, migraine headaches, and bipolar disorder. VA also appears to have anti-cancer properties with numerous possible applications, emphasizing the need for further studies either as a single agent or as an add-on therapy. The effects of VA on differentiated SH-SH5Y cell cultures appeared to be rapid, consistent, and effective, causing extensive cell detachment and cell death, which could have implications for its use in experimental or therapeutic contexts. These observations show a correlation between VA concentration and cytotoxicity, while further research is necessary to elucidate the underlying mechanisms and long-term effects, confirming causality. In addition, these observations highlight the need to re-evaluate the relevance of these literature-derived concentrations in the context of in vitro neuronal models to ensure experimental validity and safety.
